# The "Unemployed" Bill

**Published:** 1905-08-12

**Authors:** 


### The "Unemployed" Bill.

The safe passage of the Bill for the relief of the unemployed through the quicksands of Committee in the House of Commons, and the practical certainty hence arising that it will become law during the present session of Parliament, render it possible to examine its chief provisions from the point of view of their probable influence upon public health. We have, unfortunately, been rendered familiar, in recent years, with the more superficial consequences of annually recurring periods of acute distress in various industries, with the organised processions or other forms of demonstration of the unemployed, and with the parasitical adhesion to these demonstrations of a vast mass of professional vagabondage, attracted by the hope of sharing in relief funds and other forms of almsgiving. The exceedingly dangerous and noxious character of these conditions and occurrences were from the first sufficiently apparent to all who attentively regarded them, although it has only recently been thought necessary to make some legal provision against their unchecked recurrence. The efficacy of that provision has, to say the least, been imperilled by the exigencies of party politicians; but as the leaders of the opposition to the Bill have decided that the public opinion of their chief supporters would not sanction a policy of mere obstruction to a measure which was almost universally admitted to be at least better than nothing, the normal majority of the Government has produced its due effect, and the next three years will afford evidence of the results of the first attempt which has been made to deal with a great and acknowledged evil in a manner at all commensurate with its magnitude. In the passage of the Bill through Committee two great safeguards have been introduced, the requirement of a residential qualification from applicants for exceptional relief, and the prohibition of payment from the rates for the labour done in farm colonies or other eleemosynary institutions.

The decision of the Prime Minister to appoint a Royal Commission to inquire into the operation of the existing Poor Law, and to report thereupon, if possible, in time to allow of permanent legislation against the time when the Act now to be passed will expire, will afford opportunities, which we hope will be recognised and employed, for investigating the relations between poverty and disease, and for considering to what extent it may be possible to provide against what must be admitted to be their natural combination.

We have more than once pointed out that the compulsory requirement of education by the State, or, in other words, the expenditure of public money upon the children of private individuals, carries with it, as a natural consequence, the acquirement of a certain public property in these children, and a right to watch over them in such a manner as to secure, as far as may be possible, that the expenditure is not wholly wasted. The same principle, we hold, applies to the relief of poverty. Our forefathers decided, centuries ago, that our poor should have a legal claim to bare maintenance, to the absolute necessaries of life, and from this principle, even if we wished to do so, we cannot now recede. The law about to be passed will assert, in principle if not in words, that the *deserving* poor shall have a legal claim greater than this, a claim to be assisted, in times of trouble, to preserve a station from which they would otherwise be likely to fall, and the standard of desert, which even at the first will not be an exalted one, is almost certain to be lowered as time goes on. Even at the first, a man is likely to be classed as deserving who, at certain seasons of the year, has worked at his trade; even although this trade was one which, like that of builders or out-door painters, is necessarily subject to alternations of good wages and of compulsory idleness, and although the individual in question may have failed to make, during the former periods, any sort of provision for the inevitable occurrence of the latter. The intervention of the State, although for the present it be only in the form of private benevolence organised under legal sanction, to relieve him from the natural consequences of his improvidence, seems to carry with it a right to demand that his assisted household shall not, in relation to disease, become a source of public danger; and hence to imply that the local sanitary authorities should possess a dominion over him which they might not in other circumstances be justified in assuming. The right of those who confer benefits to exact conditions is one that cannot be questioned, and it is also one that, in the interests of public health and safety, should clearly be exercised by the State in the case of the unemployed.

